# Left Coronary Artery—Right Ventricle Fistula Case Report: Optimal Treatment Decision

**DOI:** 10.3390/medicina61010056

**Published:** 2025-01-02

**Authors:** Stefan Veljković, Ana Peruničić, Jovana Lakčević, Armin Šljivo, Dragana Radoičić, Mihajlo Farkić, Darko Boljević, Jelena Kljajević, Milovan Bojić, Aleksandra Nikolić

**Affiliations:** 1 Cardiovascular Institute “Dedinje”, 111040 Belgrade, Serbia; anaperunicic@hotmail.com (A.P.); jovana.lakcevic@gmail.com (J.L.); dragana.kastratovic@yahoo.com (D.R.); mihajlo.farkic@gmail.com (M.F.); darkoboljevic@gmail.com (D.B.); jelenakljajevic@gmail.com (J.K.); dedinje@ikvbd.com (M.B.); nikolicdrsasa@gmail.com (A.N.); 2 Department of Cardiosurgery, Clinical Center of University of Sarajevo, 71000 Sarajevo, Bosnia and Herzegovina; sljivo95@windowslive.com; 3 Faculty of Medicine, University of Banja Luka, 78000 Banja Luka, Bosnia and Herzegovina; 4 Faculty of Medicine, University of Belgrade, 11000 Belgrade, Serbia

**Keywords:** coronary artery fistula, multimodal imaging, congenital heart anomaly, long-term follow up

## Abstract

Coronary artery fistulas (CAFs) are rare congenital anomalies, presenting in 0.05–0.9% of cases, characterized by an aberrant connection between a coronary artery and a cardiac chamber or great vessel. Clinical manifestations can include heart failure, myocardial ischemia due to coronary steal, arrhythmias, or infective endocarditis. We report a case of a 39-year-old man initially evaluated in 2016 for peripheral edema and suspected right ventricular (RV) abnormality. Earlier assessments indicated a left anterior descending (LAD) coronary artery–RV fistula, but initial catheterization was nondiagnostic. Transthoracic echocardiography (TTE) revealed a dilated left coronary artery (LCA) and an RV apex aneurysm, confirmed by CT and coronary angiography, showing a 14 mm LAD fistula with large aneurysmal sacs (45.6 × 37.3 mm). Cardiac MRI demonstrated a tortuous LAD fistula draining into RV aneurysmal sacs with preserved biventricular function. Surgical intervention was recommended, but the patient declined and was lost to follow-up until 2022, being asymptomatic. Re-evaluation showed progression in aneurysm size (47 × 45 mm and 16 × 18 mm) without ventricular functional change. Follow-up TTE in 2023 indicated stable findings. This case emphasizes the necessity of multimodal imaging (TTE, CT, MRI, angiography) for CAF diagnosis and management planning. Given the variability in CAF presentation and outcomes, individualized management—including surgical, percutaneous, or conservative strategies—is crucial. Persistent follow-up is essential for monitoring potential complications and guiding treatment, even in asymptomatic patients refusing intervention.

## 1. Introduction

Coronary artery fistulas (CAFs) represent rare congenital cardiovascular anomalies characterized by an abnormal communication between a coronary artery and a cardiac chamber or major vessel. These fistulas are reported with an incidence ranging from 0.05% to 0.9% based on coronary angiographic findings in various large cohort studies, reflecting their uncommon occurrence [1]. The clinical presentation of CAFs is highly variable and can range from asymptomatic findings to severe complications. Symptomatic cases often arise due to hemodynamic consequences such as myocardial ischemia, which results from the phenomenon known as coronary steal—where blood flow is diverted from the coronary artery bed distal to the fistula, compromising myocardial perfusion. Heart failure symptoms can develop due to the volume overload associated with shunting, leading to ventricular dilation and dysfunction over time. Additionally, arrhythmogenic manifestations, including palpitations and syncope, may be observed due to electrical disturbances caused by altered myocardial blood flow or structural changes in the heart. Another significant clinical consideration is the potential for infective endocarditis, which can occur due to turbulent blood flow within the fistula, promoting endothelial damage and nidus for infection [2,3].

The heterogeneity of the clinical outcomes and presentations highlights the complexity of diagnosing and managing CAFs. Early identification and comprehensive assessment using a combination of noninvasive imaging modalities (such as echocardiography, computed tomography, and magnetic resonance imaging) alongside invasive techniques like coronary angiography are crucial for optimal patient management and the prevention of adverse sequelae.

## 2. Case Presentation

The patient, a 1983 birth cohort male, was initially referred to our institution in 2016, presenting with peripheral edema and a suspected morphological abnormality of the right ventricle (RV). Historical medical records indicated that, during childhood, a left anterior descending (LAD) coronary artery to RV fistula was clinically suspected. At 12 years of age, the patient underwent diagnostic cardiac catheterization (CC); however, definitive visualization of the coronary artery to RV fistula was not achieved at that time. Upon admission in 2016, the patient reported no significant family history of cardiovascular or congenital abnormalities. He described an unremarkable childhood apart from occasional episodes of fatigue during physical activity. There was no history of rheumatic fever, connective tissue disorders, or significant infections. The patient denied tobacco use, alcohol overconsumption, or substance abuse. A comprehensive review of systems was negative for chest pain, dyspnea, syncope, or palpitations. Physical examination in 2016 revealed a mildly elevated jugular venous pressure and bilateral pitting edema up to the mid-calf, with no cyanosis or clubbing. A systolic murmur (grade II/VI) was auscultated at the left parasternal border. Peripheral pulses were symmetrical, and no other significant abnormalities were noted. Initial laboratory tests performed at admission in 2016 showed the following: complete blood count: hemoglobin 14.2 g/dL, hematocrit 41.5%, white blood cells 7.3 × 10^9^/L, and platelets 246 × 10^9^/L; serum biochemistry: creatinine 0.92 mg/dL, urea 22 mg/dL, and electrolytes within normal ranges. Liver function tests: ALT 22 U/L, AST 19 U/L, and total bilirubin 0.7 mg/dL; coagulation profile: INR 1.1, aPTT 29 s; cardiac biomarkers: troponin I < 0.01 ng/m; urinalysis: no proteinuria, hematuria, or signs of infection.

**Figure 1:**
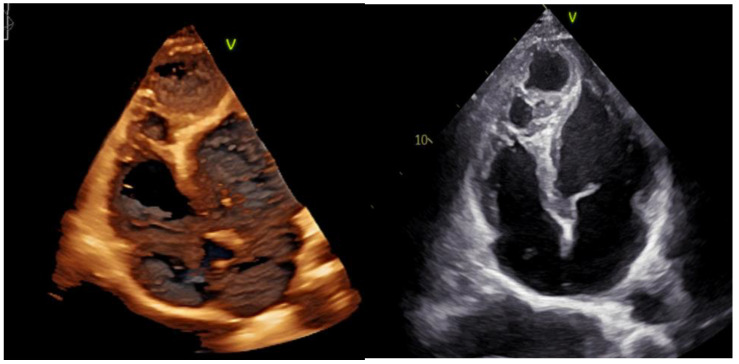
TTE: 3D (**left**) and 2D (**right**) revealing two dilated and connected aneurysmal sacs in projection of heart apex.

**Figure 2:**
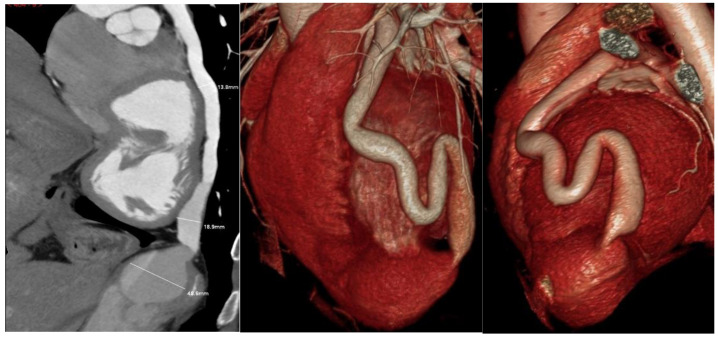
CT angiography revealing large fistula communicating with aneurysmal sac.

**Figure 3:**
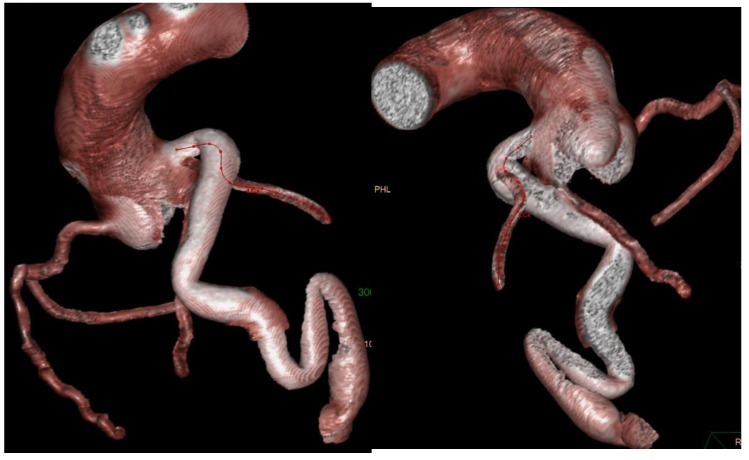
CT angiography focusing on coronary artery and fistula.

Following the initial transthoracic echocardiographic (TTE) assessment (Figure 1) at our institute, which identified a dilated origin of the left coronary artery (LCA) and an aneurysm at the RV apex, the patient was referred for computed tomography (CT) and CC, which confirmed the LCA dilatation and identified a markedly dilated LAD accompanied by a large 14 mm diameter fistula that communicated with a giant aneurysmal sac, measuring 45.6 × 37.3 mm, in the apical region of the heart (Figure 2 and Figure 3). No increase in SO_2_ levels was registered in the cardiac chambers during catheterization and coronary angiography in 2016 (SO_2_ in the left ventricle 96%; superior vena cava 71%, inferior vena cava 72%; right ventricle 71%, pulmonary artery 71%), and it was assessed that there is no significant shunt. In 2018, cardiac magnetic resonance imaging (MRI) was performed, corroborating the presence of an extensive coronary fistula originating from the ostium of the dilated LCA. The fistula demonstrated a tortuous pathway, terminating in two dilated, interconnected aneurysmal sacs near the apices of the left and right ventricles, with the distal sac draining into the RV. The MRI further demonstrated borderline enlargement in ventricular volumes, with both ventricles exhibiting preserved global contractile function. Surgical intervention was recommended following this evaluation; however, the patient declined the procedure and subsequently was lost to follow-up until 2022, when he was once again referred to our institute by his primary care physician. On examination, the patient was asymptomatic. TTE revealed an enlarged left ventricle and a borderline-sized RV, with preserved ventricular function in both chambers. Additionally, the evaluation identified a dilated origin of the LCA and two interconnected aneurysmal sacs, with the larger sac measuring 47 × 45 mm and the smaller measuring 16 × 18 mm. A repeat CT angiography confirmed these findings. At follow-up TTE in 2023, no changes were observed in LV and RV size or function, and the patient remained asymptomatic.

## 3. Discussion

This case report details a patient presenting with a congenital coronary artery fistula originating from the LAD and terminating in the RV, leading to the formation of aneurysmal sacs in the apical region of the heart. The patient initially exhibited symptoms of ankle edema alongside a suspected morphological abnormality of the RV. A comprehensive diagnostic approach, utilizing both noninvasive and invasive modalities—including TTE, CT, MRI, and coronary angiography—was employed to confirm the diagnosis. Surgical intervention was recommended, but the patient declined and was subsequently lost to follow-up, returning to our institute only after a four-year period.

The rarity of coronary artery fistulas presents a significant challenge in establishing standardized management guidelines [4,5,6]. In this case, a multimodal diagnostic approach confirmed the diagnosis, but, despite initial surgical recommendations, the patient declined and was lost to follow-up, returning after four years, highlighting the importance of sustained follow-up for patients with CAF, regardless of surgical treatment decisions.

Noninvasive diagnostic modalities, including TTE, CT, and MRI, are valuable tools for the initial assessment of coronary artery fistulas [4,5,7,8]. Invasive techniques, such as coronary angiography, are instrumental in confirming the diagnosis and delineating the anatomical extent of the fistula. Treatment for CAFs is determined by factors such as fistula size, location, and symptoms, with options ranging from percutaneous interventions like transcatheter embolization to surgical approaches including ligation, resection, CABG, or, in select cases, conservative management with surveillance [4,5,9,10,11]. Asymptomatic cases are managed conservatively with surveillance, as spontaneous closure occurs in 1–2% of the cases. Definitive closure is indicated for large or symptomatic CAFs, including those causing arrhythmia, ventricular dysfunction, or myocardial ischemia, in accordance with ESC and AHA recommendations. Accurate anatomical delineation of the CAF’s origin and termination is paramount for procedural planning and patient safety. Proximal CAFs are addressed at or near their origin to preserve myocardial perfusion, while distal CAFs are managed with occluder devices placed distally to protect proximal vascular integrity. Surgical ligation remains the modality of choice for tortuous or diminutive fistulas where endovascular access is limited. Percutaneous transcatheter closure is favored for narrow, symptomatic CAFs without concurrent cardiac anomalies, particularly in pediatric and geriatric populations. Techniques include deployment of vascular plugs, coils, or covered stents. Surgical intervention is reserved for CAFs associated with compromised myocardial perfusion, multiple fistulas, or aneurysmal dilation, often requiring ligation or bypass grafting [12].

## 4. Conclusions

The management of coronary artery fistulas necessitates a multidisciplinary approach and consistent follow-up. Employing both noninvasive and invasive diagnostic techniques is essential for accurate diagnosis and for assessing the anatomical extent of the fistula. Treatment selection is contingent upon factors such as fistula size, anatomical location, and symptomatology. Ongoing follow-up remains crucial for patients with coronary artery fistulas, including those who decline surgical intervention, to monitor potential progression and manage associated risks effectively.

## Data Availability

The original contributions presented in this study are included in the article. Further inquiries can be directed to the corresponding author(s).
